# The Association Between Cardiovascular Function, Measured as FMD and CVC, and Long-Term Aquatic Exercise in Older Adults (ACELA Study): A Cross-Sectional Study

**DOI:** 10.3389/fphys.2020.603435

**Published:** 2020-11-13

**Authors:** Markos Klonizakis, Beatrice E. Hunt, Amie Woodward

**Affiliations:** ^1^Lifestyle, Exercise and Nutrition Improvement Research Group, Sheffield Hallam University, Sheffield, United Kingdom; ^2^Centre for Sport and Exercise Science, Sheffield Hallam University, Sheffield, United Kingdom

**Keywords:** swimming, microcirculation, macrocirculation, aging, vascular

## Abstract

**Introduction:**

Cardiovascular aging is implicated in the development of cardiovascular disease (CVD). Aquatic exercise is being considered as a co-adjuvant form of rehabilitation, but there is limited evidence for its cardiovascular risk-reduction properties for older people. Our study aimed to address this by exploring the cardiovascular effects of long-term aquatic exercise in older adults in comparison to those who are either inactive or engaged in land-based/mixed training by measurement of micro- and macro-circulation. Flow Mediated Dilatation (FMD) was the primary outcome.

**Methods:**

This was a pragmatic, 4-group, cross-sectional study. Eighty normotensive adults constituted four (*n* = 20) groups. The Aqua group (aged 63.7 ± 7 years) and Land group (aged 65 ± 6 years) consisted of participants engaged in aquatic and land-based training, respectively. The mixed group (Mix) (aged 66 ± 6 years) consisted of participants engaged in both land-based and aquatic training. Self-reported training consisted of ≥2/week for ≥6 months (mean sessions/week = 4 ± 1, 4 ± 1, and 5 ± 2 for each group, respectively). The sedentary group (Sed) (aged 63 ± 6 years) consisted of people who were sedentary for ≥6 months (mean sessions/week 0 ± 0). The primary outcome was %FMD. Secondary outcomes included raw cutaneous vascular conductance (CVC) and CVC max.

**Results:**

Statistically significant differences (%FMD, raw CVC variables other than baseline) were found between each of the exercise groups (Aqua, Land, Mix) and the sedentary group (Sed) (i.e., 11.2 (4.2) vs. 5.0 (2.3); *p* < 0.0005, between the Aqua group and Sed group, for %FMD). No specific advantage could be attributed to any one of the exercise groups.

**Conclusion:**

We reported improvements in NO-mediated endothelial function at micro- and macro-circulatory levels, observing no differences between exercise modes. Our findings provide evidence for the role of aquatic exercise as a “shield” against CVD in older populations.

## Introduction

Aging affects the human cardiovascular system at both the functional and structural level. Cardiovascular aging appears to affect pathophysiological pathways also implicated being in the development of cardiovascular disease (CVD). This is why advanced age is one of the most important CVD risk factors. As almost 20% of the world population will be above the age of 65 by 2030, an exponential increase in CVD prevalence will be occurring at the same time (Heidenreich et al., 2011).

Exercise may delay cardiovascular aging and assist in CVD prevention among older individuals (Lee et al., 2012). Nevertheless, participation in exercise in older populations is low and decreases as age increases, i.e., only 13% of women over 75 meet the U.K. physical activity recommendations (Townsend et al., 2015). Fear of injury and lack of confidence in using exercise equipment are often cited as significant barriers to participation (Schutzer and Graves, 2004). Therefore, interventions based on forms of exercise that older people feel comfortable with and in which they are keen to participate, need to be promoted.

Aquatic exercise (usually swimming and/or aqua aerobics) is being considered as a potential co-adjuvant form of rehabilitation, especially for people with coronary artery disease presenting with musculoskeletal comorbidities (Cugusi et al., 2019) and peripheral arterial disease (Park et al., 2019). In older populations aquatic exercise appears to offer positive outcomes particularly in regard to gait, balance and mobility (Carroll et al., 2019).

On the other hand, there is limited high-quality evidence to support the use of aquatic exercise for improving physiological components, which are considered as risk factors for falling and physical functioning (Waller et al., 2016). Additionally, there is limited evidence to ascertain the benefit on cardiovascular outcomes for this exercise modality, for older people, while aquatic exercise studies often avoid focusing on older age-groups. Nevertheless, recent systematic reviews, mixing middle-aged and elderly participants, found that aquatic exercise reduces systolic blood pressure to levels similar to that elicited by land-based exercise (Igarashi and Nogami, 2018; Reichert et al., 2018). Furthermore, water-based exercise interventions for 12 weeks appear to offer micro- and macro-vascular benefits to older people with Type 2 Diabetes Mellitus (T2DM) (Suntraluck et al., 2017), as well as improvements in lipid measures and insulin, particularly in the longer term (i.e., following 6–12 months of training) (Cox et al., 2010). Water-aerobics performed twice a week for 12 weeks has also been indicated to improve explosive strength, body composition, and blood pressure in middle-aged adults and older adults, albeit with no concurrent improvement in lipid profile and cardiorespiratory fitness (Pereira Neiva et al., 2018).

However, as the evidence isn’t conclusive, the debate is still quite strong even for the general older adult population, with researchers questioning whether regular aquatic exercise confers beneficial effects on coronary heart disease risk factors (Tanaka, 2009) or general heart health (Lazar et al., 2013).

Our study (the ACELA study) aimed to address the knowledge gap, by exploring the cardiovascular effects of long-term (>6 months of duration) aquatic exercise (comprising either/or swimming and aquatic exercise classes) in older (>55 years of age), normotensive adults against their counterparts who were either inactive or were being trained primarily using land-based exercises or a mixture of both aquatic and land-based exercise. Our objectives were to (i) assess both micro- and macro-circulation, in order to have a complete picture of the physiological effects at both a micro- and macro-circulatory level, and (ii) to test the hypothesis that all forms of exercise offered the same cardio-protective benefits, using flow-mediated dilation (FMD) as our primary outcome.

## Materials and Methods

### Study Design

This study is reported according to the Strengthening of the Reporting of Observational Studies in Epidemiology (STROBE) guidelines (Von Elm et al., 2007). The ACELA study was a pragmatic, 4-group, cross-sectional study, approved by Sheffield Hallam University Sports Ethics Review Committee (ER5320861). All experiments were conducted in accordance with the revised Declaration of Helsinki and all participants provided written consent to participate in the study. This was a single-site, single-visit study taking place at Collegiate Hall, Collegiate Crescent Campus of Sheffield Hallam University. All assessments were undertaken in a physiology laboratory by members of the research team.

### Participants

General inclusion criteria included being over 55 years of age and normotensive (e.g., <140/90 mm Hg). For the three exercise groups, participants must have been engaging in either water-based (Aqua), land-based (Land), or water and land-based (Mix) moderate intensity, primarily aerobic-based training regimes at least 2/week for 1 h per session, for a period of at least 6 months, but less than 5 years. Participants in the sedentary group (Sed), must have been undertaking less than 60 min of structured/planned physical activity per week, for at least 6 months, but less than 5 years.

Exclusion criteria included having any overt chronic disease which would affect microvascular functioning (i.e., diabetes mellitus and coronary heart disease), anemia (irrespective of whether an iron supplementation course was followed or not), and a recent (3 months’ ago) major surgery. Participants undertaking high intensity interval training of any form were excluded. Recruitment was completed with a minimum target number per group, until group-matching was achieved.

Participants were recruited via social and mass media (Twitter, Facebook, newspaper), posters (in community venues and halls, Sheffield Hallam University, places of worship and post-offices), an open e-mail invitation and word of mouth. Participants who agreed to take part who met the inclusion criteria and agreed to take part, were invited to Collegiate Hall, Collegiate Crescent Campus of Sheffield Hallam University for a single visit, during the morning hours of the day.

### Outcome Measures

Cutaneous vascular conductance (CVC) and flow mediated dilation (FMD) and were chosen as outcome measures of macro- and micro-circulatory function because their methods are both highly regarded, non-invasive, and reproducible. Furthermore, they have been used in various studies exploring the effects of lifestyle interventions on cardiovascular function in both clinical and pre-clinical populations. Our research group is highly experienced, having led numerous studies using these to explore the effects of lifestyle interventions on the vascular function of pre-clinical and clinical populations (e.g., Tew et al., 2010; Klonizakis et al., 2017).

During the single-visit, all participants initially completed the following three questionnaires: (i) The online Q-Risk questionnaire which assesses cardiovascular disease risk (Hippisley-Cox et al., 2017), (ii) The SF-IPAQ questionnaire which assesses physical activity levels (Craig et al., 2003), and (iii) the EQ5D-5L questionnaire, to support assessment of quality of life (Herdman et al., 2011).

Participants then completed the following assessments in the order indicated below:

(a) Anthropometry: Stature, body mass, body fat (In Body 720, In Body Co., Seoul, South Korea), waist and hip circumferences were measured (Klonizakis et al., 2017). Waist circumference was measured with the participant standing with feet together, and the tape measure placed around the narrowest part of the torso, between the umbilicus and the xyphoid process. Hip circumference was measured with the participant standing as above, and the tape measure placed around the maximum circumference of the buttocks.
(b) Microcirculatory function: Participants were instructed to abstain from caffeine 2–3 h prior to the recordings, so as to eliminate the confounding effect caffeine may have produced on vasorelaxation. Skin blood flow was measured as cutaneous red blood cell flux using a Laser Doppler Flowmeter (LDF; Periflux system 5000, Perimed 122 AB, Järfälla, Sweden) (Tew et al., 2010). Local thermal hyperemia was induced using a heating disc surrounding the probe. The probe was attached to the skin on the forearm using a double-sided adhesion sticker. Recordings of the Laser Doppler signal were made using PeriSoft Windows 9.0 software (Perimed, Järfälla, Sweden). Participants were rested in a supine position in a temperature-controlled room with a constant ambient temperature of 24°C for 35 min. Heart rate and diastolic and systolic blood pressure was recorded from the left arm at 5 min intervals throughout the protocol (Dinamap Dash 2500, GE Healthcare, United States). Baseline skin blood flow data was recorded for 5 min with the local heating disc temperature set at 30°C. Following this, rapid local heating was initiated to obtain maximal vasodilation and the temperature was increased by 1°C every 10 s until 42°C was reached. This was then maintained for 30 min following, which the test was completed. For the analysis of each recording measurement phases were defined as detailed in Table 1. The LDF values were divided by the corresponding mean arterial pressure (MAP) to give the cutaneous vascular conductance (CVC) in arbitrary perfusion units (APU)⋅mm Hg. MAP was calculated from Systolic Blood Pressure (SBP) and Diastolic Blood Pressure (DBP). The data is presented as raw CVC and CVC was normalized to maximum [%CVCmax = ((CVC/CVCmax) × 100)]. The resting Heart Rate (HR) and BP of participants were calculated from the average of measured HR and BP throughout the LDF recording period.
(c) Nitric oxide-mediated, macro (arterial)- circulatory function: We used FMD as a measure of endothelium-dependent, nitric oxide (NO)-mediated, macro (arterial)- circulatory function (Klonizakis et al., 2017). We measured FMD according to the guiding principles set out by Thijssen et al. (2011). FMD was measured using a Nemio XG (Toshiba, Tokyo, Japan) ultrasound machine (software: 3.5.000; Toshiba, Tokyo, Japan). The brachial artery was imaged at a location 3–7 cm above the cubital crease to create a flow stimulus in the brachial artery, using a 12 MHz linear transducer (Toshiba, Tokyo, Japan).

**TABLE 1:** Laser doppler flowmetry measurement phase definition.

Measurement Phase	Time Point
Baseline	The arithmetical mean of the last 2 min of the first 5 min period.
Initial peak	The arithmetical mean of the highest consecutive 30 s period within the distinct initial hyperemic response.
Plateau	The arithmetical mean of the last 2 min of heating at 42°C.
Maximum	The arithmetical mean of the last 2 min of heating at 44°C.

Baseline scanning to assess resting vessel diameter was recorded over 3 min, following a 10 min resting period. A rapid inflation-deflation pneumatic cuff placed immediately distal to the elbow joint was used to as an FMD stimulus. The cuff was inflated to 200 mm Hg for 5 min, with recordings commencing 30 s before cuff deflation and continuing for 3 min after. Percentage FMD (FMD%) was the measure of assessment, being calculated by dividing the change in arterial diameter in the mean cardiac phase by the initial baseline diameter of the artery and multiplying it by 100. After the assessment, participants were then monitored for an additional 5 min before the study visit was completed.

### Bias and Study Size

Evaluators were blind to participant grouping to reduce investigator bias. Additionally, to decrease inter-observer bias and increase measurement consistency, each evaluator carried out the same measurement for each participant. That is, one undertook the microcirculatory assessment each time while another undertook the macro-circulatory assessment each time.

The sample size was achieved by setting a minimum target number per group (i.e., *n* = 18). Once this was met, participants were recruited, until group-matching was achieved.

### Data Analysis

Normality of each of the outcome variables was assessed using the Shapiro-Wilks test. All variables were non-parametric, with the exception of MAP, CVCmax and %CVCmax baseline, which were parametric. For these 3 parametric variables, the one-way ANOVA was performed. For non-parametric variables the Kruskal-Wallis test was applied to test for overall between-groups differences. Variables showing significant differences were further analyzed for pairwise differences using the Mann-Whitney *U*-test. To avoid committing a Type I error, we used a Bonferroni correction for all between-groups analyses. Statistical analysis was performed using SPSS (IBM SPSS Statistics Version 26 (IBM United Kingdom Limited, Hampshire, United Kingdom). Statistical significance for the test was set at *p* ≤ 0.05. Values are presented as mean ± standard deviation (*SD*), unless otherwise stated.

## Results

### Demographics

**FIGURE 1:**
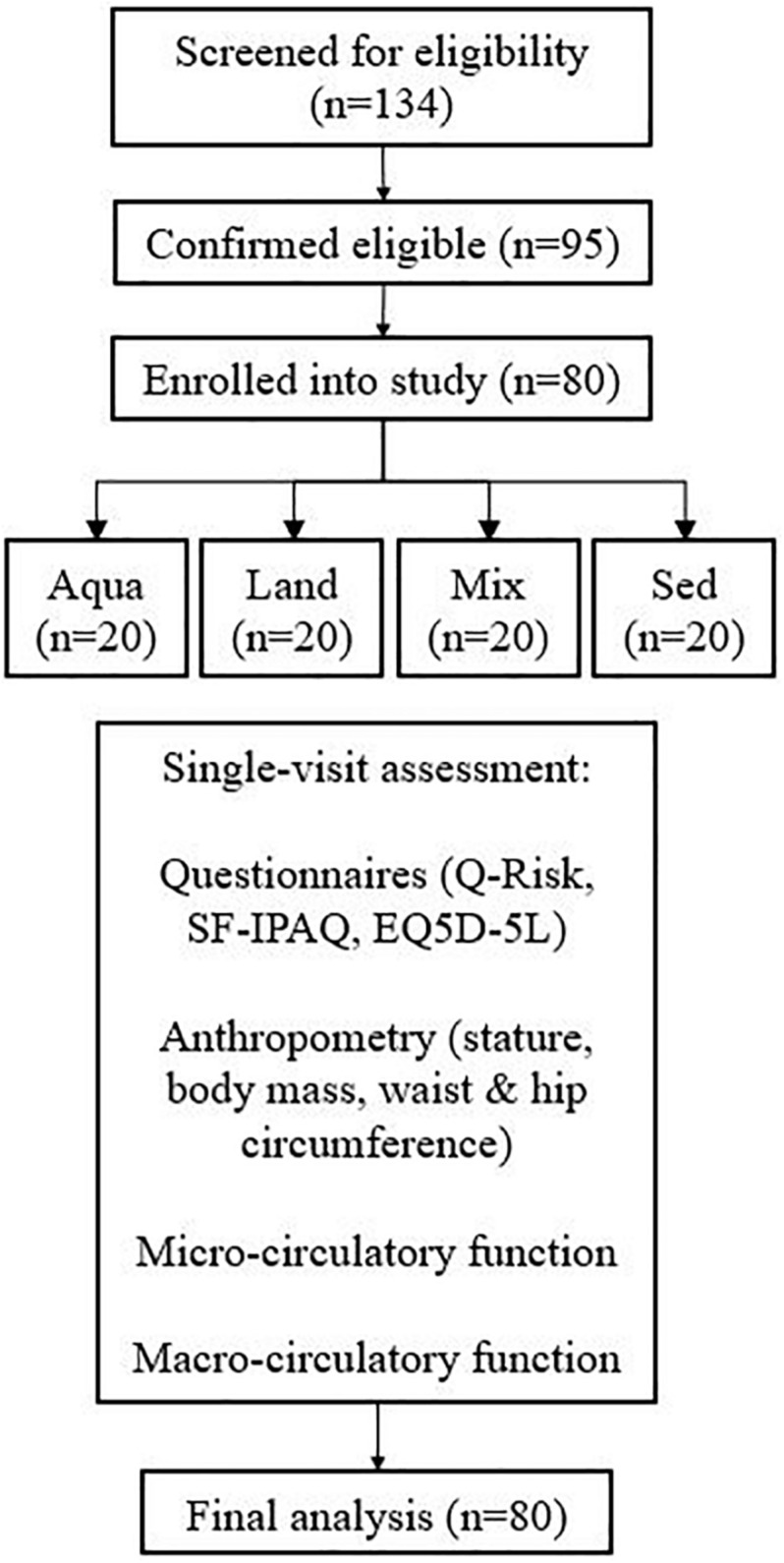
A flow diagram of the participant pathway through the study.

In total, 134 people were examined for eligibility, 95 were confirmed eligible, and eighty participants were enrolled in the study and assessed. There were 20 participants in each group. Figure 1 demonstrates the participants’ pathway through the study. Demographics are presented in Table 2. All four study groups were statistically matched for all variables with the exception of body fat and training-related variables (e.g., IPAQ-derived variables and training sessions), where pairwise comparisons, confirm the a statistically significant difference between the exercise groups and the sedentary one (e.g., body fat; Aqua vs. Sed – *p* = 0.03, Mix vs. Sed – *p* = 0.04, Land vs. Sed – *p* = 0.04). No statistically significant differences were observed between the exercise groups.

**TABLE 2:** Demographics for participants.

	Aqua (Aquatic)	Land (Land-based)	Mix (Mixed)	Sed (Sedentary)
Sex	8 M/12 F	9 M/11 F	8 M/12 F	9 M/11 F
Age	63 ± 7	65 ± 6	66 ± 6	63 ± 6
Weight (Kg)	75 ± 13	71 ± 12	75 ± 14	77 ± 16
Height (m)	1.72 ± 0.1	1.69 ± 0.1	1.69 ± 0.1	1.67 ± 0.1
BMI (kg⋅m^2^)	25.2 ± 4.2	24.7 ± 3.7	26.4 ± 4.4	27.1 ± 4.8
Waist circumference (cm)	90 ± 12	87 ± 11	93 ± 11	93 ± 12
Hip circumference (cm)	102 ± 9	100 ± 7	105 ± 11	103 ± 9
Waist to hip ratio	0.88 ± 0.11	0.87 ± 0.07	0.88 ± 0.07	0.91 ± 0.11
Body fat (%)	28.7 ± 9	27.9 ± 11	29.6 ± 8	31.7 ± 8*
Heart rate (bmp)	62 ± 11	63 ± 14	61 ± 10	67 ± 14
Systolic BP (mm Hg)	128 ± 20	129 ± 20	132 ± 22	132 ± 12
Diastolic BP (mm Hg)	78 ± 11	78 ± 8	77 ± 11	77 ± 13
MAP (mm Hg)	99 ± 14	102 ± 13	104 ± 7	98 ± 13
EQ5D-5L Visual analog scale (VAS) score	82 ± 7	81 ± 14	75 ± 14	78 ± 16
IPAQ (METs/week)	4,990 ± 2445	6,311 ± 3741	5,509 ± 3847	1,141 ± 406*
IPAQ (categorical score)	3 ± 0	3 ± 0	3 ± 0	1 ± 1
IPAQ- sitting time				
(Mins/week)	1,934 ± 888	1,529 ± 915	1,763 ± 777	3,955 ± 902*
(Mins/day)	276 ± 127	218 ± 131	252 ± 111	565 ± 128*
Q-risk (%)	10 ± 7	12 ± 6	12 ± 7	14 ± 5
Training sessions/week	4 ± 1	4 ± 1	4 ± 2	0 ± 0*

Values are expressed as mean ± standard deviation; *statistical significance = p < 0.05. M, male; F, female; Kg, kilograms; BMI, body mass index; cm, centimeters; bpm, beats per minute; BP, blood pressure; mmHg, millimeters of mercury; MAP, mean arterial pressure; VAS, visual analog scale; MET, metabolic equivalent of task.

### Micro- and Macro-Circulation

Statistically significant differences were found for all raw CVC variables, with the expected exception of CVC, with exercise groups experiencing higher values than the sedentary group (Table 3). Standardized CVC values (against CVC max), were not statistically significant.

**TABLE 3:** Between-groups comparison of cardiovascular function of older adults.

	Group				*p*-value
	Aqua	Land	Mix	Sed	
**Raw CVC (APU/mm Hg)**					
Baseline	0.6 ± 0.4	0.4 ± 0.4	0.4 ± 0.6	0.3 ± 0.1	0.2
Initial peak	3.1 ± 0.9	2.7 ± 0.9	3.2 ± 1.2	2.2 ± 0.7	0.004*
Plateau	3.7 ± 1.3	3.5 ± 1.6	3.6 ± 1.2	2.6 ± 1.1	0.005*
Max	4.4 ± 1.3	4.0 ± 1.2	4.3 ± 1.3	3.1 ± 0.8	0.002*
**CVC max (%)**					
Baseline	12.4 ± 7.3	11 ± 11.7	10.5 ± 15.5	12.5 ± 8.5	0.1
Initial peak	73.4 ± 18.7	71.3 ± 27.4	76.4 ± 20.5	71.7 ± 17.3	0.9
Plateau	84.5 ± 15.9	90.3 ± 40	85.3 ± 18.8	83.9 ± 19.7	0.9
**FMD**					
FMD (%)	11.2 ± 4.2	11.7 ± 5.2	11.4 ± 4.4	5.0 ± 2.3	<0.0005*
FMD pre-occlusion (cm)	3.98 (0.67)	4.01 (0.69)	4.02 (0.45)	3.92 (0.32)	0.6
FMD post-occlusion (cm)	4.46 (0.69)	4.45 (0.56)	4.42 (0.51)	4.12 (0.34)	<0.005*

Values are expressed as mean ± standard deviation; *Statistical significance = p < 0.05. CVC, cutaneous vascular conductance; APU, arbitrary perfusion units; CVC max, percentage of maximum CVC; FMD, flow mediated dilation. Aqua, aquatic exercise; Land, land-based exercise; Mix, mixed aquatic and land-based exercise; Sed, sedentary.

Similarly, for %FMD, there was a statistically significant difference between groups, with exercise groups having greater values than the sedentary group (Table 3).

### Pairwise Comparisons

All variables were analyzed for pairwise comparisons (Table 4) with the exception of raw CVC baseline and %CVCmax standardized ones, as these were found to be non-significant, during the “between-groups” comparison (presented in Table 3). No statistically-significant differences were detected during the pairwise comparisons between exercise groups (Aqua vs. Land, Aqua vs. Mix, Land vs. Mix; Table 4). Statistical significance (in %FMD and most of the raw CVC variables) was reached between each of the exercise groups and the sedentary group (Table 4).

**TABLE 4:** Between-groups pairwise comparisons of cardiovascular function of older adults.

	*p*-value					
	Aqua vs. Land	Aqua vs. Mix	Aqua vs. Sed	Land vs. Mix	Land vs. Sed	Mix vs. Sed
**CVC**						
Initial peak	0.10	1.00	0.001*	0.10	0.20	0.009*
Plateau	0.40	0.60	<0.0005*	0.50	0.05*	0.003*
Max	0.30	0.60	0.001*	0.30	0.02*	0.002*
%FMD	0.20	0.90	<0.0005*	0.40	<0.0005*	<0.0005*

*Statistical significance = p < 0.05. CVC, cutaneous vascular conductance; FMD, flow mediated dilation. Aqua, aquatic exercise; Land, land-based exercise; Mix, mixed aquatic and land-based exercise; Sed, sedentary.

## Discussion

Our study contributes to research exploring the association of long-term aquatic exercise and the cardiovascular function of “older-but-otherwise healthy” adults, who train regularly (e.g., at least twice a week), against other modes of exercise. We studied and assessed two areas of our circulation, micro- and macro-circulation; our findings in both areas suggest that regular aquatic exercise draws similar CVD risk-reduction benefits to both regular, non-high-intensity interval training, land-based exercise, as well as to a mixture of both land- and water-based exercise. However, since this was a single-visit cross-sectional study, assumptions about the causality cannot be made. Nevertheless, this observational data could help the current debate about the cardiovascular effects of aquatic exercise in older groups, particularly as our study, avoiding high-intensity interval training exercise, focuses on the where the exercise regime takes place.

Although microcirculation is considered as an important element of our circulatory system (Klonizakis et al., 2017), it is often overlooked, when designing and executing studies exploring the CVD-risk reduction properties of different interventions. This is an incorrect approach, as it is now accepted that pathological changes in the microcirculation mirror (Debbabi et al., 2010) if not precede (Lenasi, 2016) changes in arteries, therefore, making it the most appropriate region to study, to facilitate early detection.

In our study, we explored both axon reflex- and endothelium-mediated microcirculatory vasodilation, both of which are affected by aging (Tew et al., 2010), with the former being related to the body’s response mechanism to local tissue trauma (Tew et al., 2010) and the latter being considered an early sign of atherosclerosis and cardiovascular disease (Versari et al., 2009). This was conducted through the assessment of “initial peak” and “plateau” heating-response phases, respectively. In microcirculation, the “initial peak” phase during local heating relies predominantly on local sensory nerves (Minson et al., 2001) and is mediated by the axon reflex that is dependent on calcitonin-gene-related peptide and substance P (Holowatz et al., 2007), while the “plateau” phase is mediated primarily by endothelium-dependent NO release (Minson et al., 2001).

It is accepted that land-based exercise regimes offer important, microvascular CVD risk-reduction benefits (Lanting et al., 2017). Our findings suggest that these benefits extend also to regular aquatic-based exercise whether this is conducted alone or in combination with land-based exercises. The mechanism to which aquatic exercise improves microvascular function, and particularly NO bioavailability that is of primary interest in CVD, is not clear, although it is likely that this is due to an increased plasma concentration of nitrates and not catecholamine, as it has been observed in clinical populations following swimming (Mourot et al., 2009), in a manner similar to that caused following land-based exercise training (Tew et al., 2010). Nevertheless, the possibility of this occurring through a shear stress-mediated increase in endothelial NO synthase e(NOS) gene expression (Hodges et al., 2007) or via training-related changes in microvascular function (Tew et al., 2010) can also not be excluded: For example, it is known that. eNOS-generated NO facilitates dynamic alterations in blood flow distribution (e.g., in response to altered shear stress) and has antiatherosclerotic effects at the level of the endothelium (Melikian et al., 2009). Therefore, it would be useful for future studies in the field, to conduct specific experiments that will explore the exact mechanisms involved. In any case, our findings, in agreement with others (Suntraluck et al., 2017) suggest that an additional option might exist for treatment and rehabilitation purposes, for the conditions that have a strong microcirculatory element and for which aerobic exercise might be beneficial.

Most of the CVD-related studies conducted on the effects of aquatic exercise on older people, have been focused on people with coronary heart disease and heart failure (Mourot et al., 2009; Cugusi et al., 2019; Vasić et al., 2019), with little work having been completed on the longer-term effects on a general, “older-but-healthy” population (Igarashi and Nogami, 2018). This is an important knowledge gap, considering that a number of researchers debate the positive cardio-protective influence that aquatic exercise may have on our bodies (Tanaka, 2009; Lazar et al., 2013). In that sense, a study like ours was required, in order to explore cardiovascular differences in “older-but-healthy” individuals, who train regularly for a long-period of time.

Our study indicates that long-term, water-based exercise in older individuals can offer improvements in macrovascular function parameters, which are associated with CVD risk reduction, such as %FMD (Klonizakis et al., 2017). This happens in a manner similar to what is observed in the short-term, in clinical populations such as people with coronary heart disease (Vasić et al., 2019) and osteoarthritis (Alkatan et al., 2016). Our work extends the findings of other research groups on clinical (e.g., peripheral artery disease) (Park et al., 2019) and general (Sherlock et al., 2014; Reichert et al., 2018) populations, which have used alternative measures known to be related to CVD risk, such as arterial stiffness (assessed by Pulse Wave Velocity) (Sherlock et al., 2014) and blood pressure (Reichert et al., 2018), suggesting notable risk reductions, similar to that observed following land-based exercise (Di Francescomarino et al., 2009). Indeed, aquatic exercise may confer additional benefit for older adults, where participants can obtain the advantages of regular land-based exercise (i.e., improved strength, balance, flexibility, and cardiovascular health benefits) without pain, stress, or strain on joints (Heyneman and Premo, 1992; Kim and O’Sullivan, 2013).

Exploring the mechanism that triggers these positive findings, is less straight forward. In our study all participants were normotensive, and no differences were observed in MAP between groups. Thus, it is unlikely that a reduction in distending pressure contributed to the improved macrovascular function. This observation is in agreement with findings from other studies, which suggest that even when improvements are detected in arterial stiffness, these are most likely related to a reversal of endothelial dysfunction (Sherlock et al., 2014). Nevertheless, this is no surprise, looking closely to our findings: FMD, in which we observed the biggest differences between older people following aquatic exercise regimes and sedentary older adults, is known to be endothelium-dependent, NO-mediated. Considering that longer-term aquatic exercise is associated with a reduction in the markers of low-grade inflammation (Alkatan et al., 2016), the most plausible mechanism behind the observed increase in NO bioavailability, is that this was achieved through a reversal of the inflammation affected endothelial dysfunction, possible in combination with hemodynamic changes triggered by long-term aquatic exercise (Vasić et al., 2019). As we observed similar differences at microcirculatory level, the effect is most likely happening on a systemic manner, which is a novel finding worthy of further investigation (and confirmation) in future studies, exploring both micro- and macro-circulation.

Finally, the fact that all of our study participants were engaging in regular exercise (4 times per week on average), should not be overlooked: it is known that regular moderate physical activity promotes an antioxidant state and preserves endothelial function, eliciting systemic molecular pathways connected with angiogenesis and chronic anti-inflammatory action (Di Francescomarino et al., 2009) Our work suggests that irrespective of the mechanisms involved, benefits are similar between land-, water- and mixed-mode based, aerobic exercise regimes, provided that a training program is followed regularly.

### Limitations

As the study is a cross-sectional observational study, our findings cannot be used to establish a causal relationship. However, this study can be built upon to investigate causality in further research. In addition, data regarding exercise intensity and frequency is necessarily self-reported, and thus may contain some inaccuracies based on recall. We did not differentiate between alternative sub-modes of land- or water- exercise programs (e.g., aquarobics, pool swimming). Although this might be considered as a limitation, we did that purposefully as it is unlikely that people, particularly older, would undertake a single mode of exercise when joining a swimming pool, particularly when undertaking water-based exercises. They would be more likely to combine swimming and water-based exercise classes within the same week, rather than following solely one of these. Therefore, we chose to follow a pragmatic approach, rather than a traditional “laboratory study” one. We also excluded people who were not normotensive, knowing that as older age advances, hypertension is common. This was done to allow for a better comparison between participants, hoping that the next step would be to conduct larger studies in clinical populations. We also concentrated on aerobic, non-high-intensity regimes. Again, this happened in order to limit variation. Due to the fact that the study had limited funding, we were unable to explore the differences between groups on inflammation markers. Considering that the changes to that level may occur at a later point in exercise life (Alkatan et al., 2016), it would have added to our findings. In addition, due to limited funds, automatic vessel measurement software (i.e., wall tracking and edge detection) was not available, but computer-assisted measurements were made nonetheless. Finally, results were not adjusted to account for BMI or body composition variables. Nevertheless, the novelty of our work is not diminished, and our observations contribute to existing research in the field.

## Conclusion

The ACELA study is the first to compare the micro- and macro-circulatory function in groups of “older-but-healthy” adults undertaking different modes of long-term, aerobic exercise regimes. We reported improved NO-mediated, endothelial function at both micro- and macro-circulatory levels for all training groups in comparison to the sedentary group observing no differences between training environments. These findings suggest that aquatic exercise could have similar CVD risk reduction benefits to land-based exercise in this population. Although this study is a cross-sectional study and outcomes were not measured over time, our findings are in agreement with work by others (Sherlock et al., 2014; Cugusi et al., 2019; Vasić et al., 2019). This study provides support for the CVD risk-reduction role of aquatic exercise and highlights a potential next step, which would be developing a cohort study to measure changes in circulatory function over time.

## Data Availability Statement

The datasets presented in this article are not readily available because Ethical approval precludes sharing of datasets. Requests to access the datasets should be directed to Dr. Markos Klonizakis, m.klonizakis@shu.ac.uk.

## Ethics Statement

The studies involving human participants were reviewed and approved by the Sheffield Hallam University Sports Ethics Review Committee (ER5320861). The patients/participants provided their written informed consent to participate in this study.

## Author Contributions

MK and BH conducted the recruitment. MK, BH, and AW conducted the testing and wrote the manuscript. All authors contributed to the article and approved the submitted version.

## Conflict of Interest

The authors declare that the research was conducted in the absence of any commercial or financial relationships that could be construed as a potential conflict of interest.
